# ASO Author Reflections: Breast Cancer-Related Lymphedema Prevention: The Impact of Prophylactic Lymphovenous Bypass

**DOI:** 10.1245/s10434-024-16824-2

**Published:** 2025-01-12

**Authors:** Min-Jeong Cho, Sydney Chratian, Roman Skoracki

**Affiliations:** Department of Plastic and Reconstructive Surgery, The Ohio State University, Columbus, OH USA

## PAST

Breast cancer-related lymphedema (BCRL) is a debilitating complication of axillary lymph node dissection (ALND), affecting up to 50% of patients and leading to significant physical, emotional, and financial burden.^1^ Microsurgical procedures such as a lymphedema microsurgical preventive healing approach (LYMPHA) and prophylactic lymphovenous bypass (pLVB) are performed at the time of ALND to prevent BCRL by reconnecting disrupted lymphatic vessels to neighboring veins, effectively restoring lymphatic flow.^2–4^ Although these procedures have demonstrated promising results in decreasing lymphedema risk after ALND, literature evaluating the long-term outcomes of ALND with and without pLVB remains limited.

## PRESENT

This study retrospectively reviewed 278 patients who underwent ALND alone and 92 patients who underwent ALND and pLVB. The study found that pLVB significantly reduced the incidence of BCRL compared with ALND alone (8.7% vs. 20.1%).^5^ Additionally, patients treated with ALND and pLVB demonstrated significant improvements in postoperative circumferential measurements and reported fewer positive symptoms of lymphedema such as pain, tightness, and heaviness. For those who experienced BCRL despite ALND and pLVB, disease severity was mitigated, with lower rates of cellulitis and reduced need for therapeutic surgery. These findings highlight the role of pLVB in enhancing both the physiologic and psychosocial outcomes for breast cancer patients undergoing ALND, emphasizing its value in comprehensive cancer care.

## FUTURE

The results of this study support the broader implementation of pLVB as a safe and effective approach for at-risk patients undergoing ALND. Prophylactic lymphatic reconstruction plays a crucial role not only in preventing BCRL, but also in reducing the disease severity for patients who experience BCRL despite intervention. Further research is needed in larger-scale studies to validate these findings, further refine patient selection criteria, and comprehensively assess the clinical impact of pLVB.
